# Sense of Coherence (SOC) Among Psychotherapists in Austria, Differentiated According to Number of Individually Completed Training Therapy Sessions

**DOI:** 10.1100/tsw.2006.349

**Published:** 2006-10-02

**Authors:** Heinz P. Binder, Elke Mesenholl-Strehler, Paul Paß, P. Christian Endler

**Affiliations:** Interuniversity College for Health and Development, Castle of Seggau, Austria

**Keywords:** sense of coherence, SOC, Antonovsky, psychotherapists, training therapy, selfawareness sessions, Austria

## Abstract

The sense of coherence (according Aaron Antonovsky, 1923—1994, when a persons sense that his/her own life and the world are sufficiently comprehensible, manageable, and meaningful) of Austrian psychotherapists was assessed and compared with a standard sample, as well as with the sense of coherence (SOC) of members of other professions. In addition, the question as to whether psychotherapists who had completed more extensive individual training therapy/self-awareness sessions had a higher SOC than do those with fewer, was addressed. Forty psychotherapists who worked in private practices and various psychosocial health care institutions in Styria, Austria took part in the study. The investigation was conducted in the form of a questionnaire assessment. The evaluation showed that the overall SOC value of the professional group in question was significantly higher than that of the standard sample (162.3 vs. 145.7), as well as other samples (physicians: SOC = 153.8; teachers: SOC = 156.1; physiotherapists SOC = 158.1). Concerning whether psychotherapists who had completed more individual training therapy/self-awareness sessions had higher SOC values than did those with fewer, we found no difference in regard to the overall SOC score or SOC scores for individual components. The SOC of psychotherapists did not seem to depend on the number of additional training therapy/self-awareness sessions.

## INTRODUCTION

Psychotherapists are subject to identity-relevant stress factors to an exceptional degree. In their work they: (a) engage in the emotional dimension of human beings — wishes, desires, and aggressions; (b) are constantly exposed to the many different qualities of emotional disorders; and (c) must patiently help others to rediscover time-tested resources and implement new strategies for mastering life situations. The emotional resiliency of these psychotherapists must be such as to allow them to fulfill all these tasks satisfactorily.

According to Aaron Antonovsky (1923–1994)[1,2] a person's sense that his/her own life and the world around them are sufficiently comprehensible, manageable, and meaningful was described as the sense of coherence (SOC), which stands in direct connection with that person's capacity to meet the demands that are made on him/her. The SOC questionnaire developed by Antonovsky aimed to measure this SOC[3,4].

This study was concerned with the degree to which psychotherapists in Styria, Austria possessed such SOC. First of all, the SOC of the psychotherapists was assessed and compared to a standard sample from the literature[5], as well as with the SOC of members of other professions. In addition, we wanted to know if psychotherapists who had completed more extensive individual training therapy/self-awareness sessions had a higher SOC than others.

## METHODS

The investigation at hand constitutes a quantitative data collection based on one single assessment using a standardized SOC questionnaire as developed by Antonovsky[3,4], as well as a demographic questionnaire.

Forty psychotherapists who worked in private practices and at various psychosocial health care institutions in Styria, Austria took part in the study. This study was conducted in the form of a questionnaire assessment. The questionnaire was sent out via the Internet (e-mail) with the network for psychotherapy in Styria acting as distributor. The questionnaire was mailed to a total of 210 persons. The accompanying e-mail expressly stated that the collected data would be treated confidentially and that no person-related evaluations would be carried out. Most of the questionnaires were returned via e-mail, while others were mailed or faxed. In addition, those on staff at institutions had the option of returning their filled-out questionnaires collectively.

The replies were examined using main component analysis. In the course of the evaluation, subgroups were formed according to demographic variables as well as the number of completed training therapy/self-awareness sessions. Univariate variance analyses (ANOVA) were conducted. Furthermore, post-hoc tests (Scheffé procedure) were performed. In keeping with conventional application, the significance level was defined as $p \leq 0.05$ for all calculations. Standard tests on correlations and the homogeneity of variances were also performed.

## RESULTS

The evaluation showed that the overall SOC value of the professional group in question was 162.3. This shows that the psychotherapists evidenced a significantly higher overall SOC value than that of the standard sample (average population, Germany)[5] (145.7), as well as other samples (physicians: SOC = 153.8[6]; teachers: SOC = 156.1[7]; physiotherapists SOC = 158.1[8]).

Concerning the question as to whether psychotherapists who had completed more individual training therapy/self-awareness sessions had higher SOC values than did those with fewer, the results showed no difference in regard to the overall SOC score or SOC scores for individual components. The SOC of psychotherapists did not seem to depend on the number of additional training therapy/self-awareness sessions (see Table 1).

## DISCUSSION

The relatively high SOC value calculated for the psychotherapists who participated in the study (162.3)[10] was not surprising when compared with the results of members of other helping professions[6,8]. On the one hand, this high value might possibly be explained by the selection of persons who choose a helping

**TABLE 1**
**SOC, Dependent on Number of Individually Completed Training Therapy Sessions (TS)**

| SOC | TS |
|---|---|
| 162.4 | ca. 130 h |
| 164.2 | 130–190 h |
| 160.0 | > 190 h |

profession and are capable of practicing it long term. On the other hand, it may be attributable to the image that members of helping professions have of themselves or like to convey. Moreover, one should note that the response quota for the questionnaire was relatively low (40 out of 210), so this constitutes a certain preliminary selection.

The study at hand confirms Antonovsky's hypothesis that SOC is a "trait variable", i.e., a factor independent of time and treatment. It shows itself to be independent of the number of individually completed training therapy/self-awareness sessions.

According to Antonovsky, the development of SOC is consolidated by early adulthood. Initially, he expressed skepticism with regard to possibilities for influencing it psychotherapeutically. As he wrote: "it is utopian to expect that an encounter or a series of encounters between the client and a therapist could effect any significant change in the sense of coherence." He deemed that a slight change of the SOC value measured by the questionnaire within a range of 5 points could be achieved through therapeutic intervention[2].

The investigations carried out by Sandell[9,10] showed a clear increase in SOC through long-term psychoanalytic therapy. In this comparative Scandinavian study, 209 patients in three different treatment settings were evaluated: (a) classic psychoanalytic setting with four sessions a week; (b) intensive psychotherapy with two to three sessions a week; (c) less-intensive psychotherapy with up to 1 h/week. The results showed that SOC stayed more or less the same for those with less-intensive treatment, whereas intensive psychotherapy led to a slight or medium increase in SOC and classic analysis effected the most pronounced, sustained improvement.

The SOC values for the psychotherapists evaluated in the present study did not differ in terms of the number of individually completed training therapy/self-awareness sessions. The contrast with the study conducted by Sandell[9,10] could be attributed to the fact that the psychotherapists' SOC values were higher from the very beginning as compared to those of Sandell's patient group, meaning that the study dealt with a population that had more emotional resiliency from the very start. In this sense, one might also add that in keeping with Austrian legislature, psychotherapists are required to complete a relatively large number of high-quality training therapy/self-awareness sessions before they are allowed to practice their profession. Should such training have influenced their SOC from the very start, then apparently the value cannot be improved by additional or advanced training. Of course, one cannot conclude from this that additional individual training therapy or self-awareness sessions are not capable of promoting other, equally important qualities and competencies for psychotherapists. On the contrary, it could even be that further psychotherapeutic training would correct an initially unrealistic self-image and an exaggerated self-assessment in terms of SOC. Further studies aimed to examine these aspects are now being carried out.
